# Early prediction of upper limb functioning after stroke using clinical bedside assessments: a prospective longitudinal study

**DOI:** 10.1038/s41598-022-26585-1

**Published:** 2022-12-21

**Authors:** Margit Alt Murphy, Ahmad Al-Shallawi, Katharina S. Sunnerhagen, Anand Pandyan

**Affiliations:** 1 Department of Clinical Neuroscience, Rehabilitation Medicine, Institute of Neuroscience and Physiology, Sahlgrenska Academy, University of Gothenburg, Gothenburg, Sweden; 2 Department of Occupational Therapy and Physiotherapy, Sahlgrenska University Hospital, Gothenburg, Sweden; 3 The Administrative Technical College of Mosul, Northern Technical University, Mosul, Nineveh Iraq; 4 Faculty of Health and Social Science, Bournemouth University, Bournemouth, UK

**Keywords:** Stroke, Predictive markers

## Abstract

Early and accurate prediction of recovery is needed to assist treatment planning and inform patient selection in clinical trials. This study aimed to develop a prediction algorithm using a set of simple early clinical bedside measures to predict upper limb capacity at 3-months post-stroke. A secondary analysis of Stroke Arm Longitudinal Study at Gothenburg University (SALGOT) included 94 adults (mean age 68 years) with upper limb impairment admitted to stroke unit). Cluster analysis was used to define the endpoint outcome strata according to the 3-months Action Research Arm Test (ARAT) scores. Modelling was carried out in a training (70%) and testing set (30%) using traditional logistic regression, random forest models. The final algorithm included 3 simple bedside tests performed 3-days post stroke: ability to grasp, to produce any measurable grip strength and abduct/elevate shoulder. An 86–94% model sensitivity, specificity and accuracy was reached for differentiation between poor, limited and good outcome. Additional measurement of grip strength at 4 weeks post-stroke and haemorrhagic stroke explained the underestimated classifications. External validation of the model is recommended. Simple bedside assessments have advantages over more lengthy and complex assessments and could thereby be integrated into routine clinical practice to aid therapy decisions, guide patient selection in clinical trials and used in data registries.

## Introduction

Early and accurate prediction of post-stroke recovery potential, ideally during the first week, is needed to assist selection of treatment approaches and inform patient selection in clinical trials^1^. A range of models to predict upper limb motor outcome have been published^2–6^. At the simplest level, measurable grip strength at 1 month^5^ or presence of shoulder abduction or finger extension at 3-days post stroke^7^ are suggested as plausible predictors for upper limb recovery. The more complex models propose various neurophysiological and neuroimaging techniques combined with clinical assessments^8–10^.

There are, however, significant barriers to the clinical implementation of existing prediction algorithms. For example, use of more comprehensive scales, such as Fugl-Meyer Assessment, within days of stroke is a challenge in acute settings, particularly in patients with complex needs. The requirement to take repeated measurements within the first weeks^11^ can also be problematic when typical length of stay within a stroke unit is less than 2-weeks^12,13^. Further, the need to draw upon neurophysiological and neuroimaging techniques to determine the corticospinal integrity are costly and not accessible to most clinical practices^1,14^. Prediction models using easily accessible clinical data i.e. simple clinical tests with routinely available equipment early after stroke^5,6,15^ would be a more realistic solution for developing clinically usable prognostic algorithms.

Prediction models only including clinical assessments have shown to be inferior compared to the models combining clinical and neurophysiological or neuroimaging techniques^9,16,17^. This seem to be particularly valid for patients with initial poor motor function^9,16,17^. Recently, a prediction algorithm only including clinical bedside assessment reported an overall accuracy of 61% at predicting upper limb activity capacity at 3 months post stroke, although the sensitivity and specificity varied across the four outcome categories^17^. An external validation of a prediction model for upper limb activity capacity at six months post stroke, discriminating poor outcome (Action Research Arm Test < 10) and using shoulder abduction and finger extension as clinical predictor variables, showed high sensitivity (> 0.80) but lower specificity (0.40–0.70)^18^. For discrimination of a higher outcome level (Action Research Arm Test > 32), likewise, the sensitivity was high (> 0.92) but specificity was lower (0.28–0.60)^18^. These studies confirm that prediction models only using clinical assessments can provide clinically useful information, perform better than a chance alone and could therefore be considered as alternatives for more complex models^17^. Based on previous literature, to reach clinical relevance, a prediction algorithm only including clinical assessments should be expected reach an accuracy, sensitivity and specificity at least between 60 and 70%.

To target this clinical need we aimed to identify a set of clinical assessments feasible in routine practice early after stroke that can provide an accurate and differentiated prediction of upper limb activity capacity at 3-months post-stroke.

## Methods

### Participants

**Figure 1:**
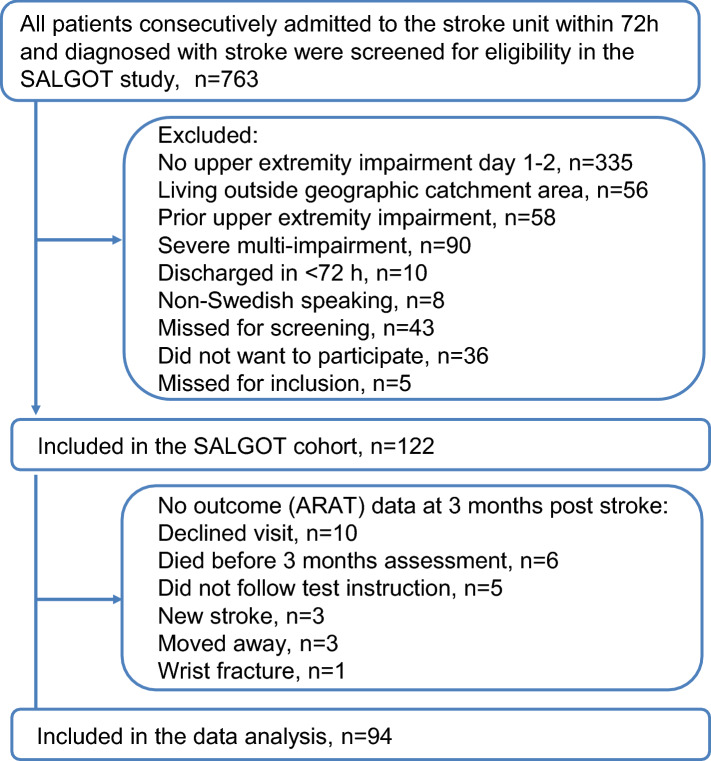
Flowchart over the inclusion process for the study group. *SALGOT* Stroke Arm Longitudinal Study at Gothenburg University.

This study was a secondary analysis of data collected in the Stroke Arm Longitudinal Study at Gothenburg University (SALGOT, https://ClinicalTrials.gov, identifier: NCT01115348), a prospective longitudinal observational study with repeated measurements, aiming to describe the recovery of upper limb functioning during the first year after stroke^19^. The SALGOT cohort comprised a non-selected population of 122 adults with first-ever clinical stroke admitted to the stroke unit within 3-days of stroke onset (Fig. 1). Patients with impaired upper limb function verified with a score below 66 of the Fugl-Meyer Upper Extremity Assessment (FMA-UE) or a score below 57 of the Action Research Arm Test (ARAT) 3-days post stroke were included. The diagnosis of stroke was based on World Health Organization (WHO) collaborative study criteria (ischemic infarct and haemorrhagic)^20^. The exclusion criteria were injury or condition prior to the stroke that limited the use of the affected arm, severe multi-impairment, diminished physical condition prior to stroke or short life expectancy (e.g. late stage of cancer, renal-disease), and not able to communicate in Swedish.

All patients received multi-professional team-based rehabilitation according to the Swedish National Guidelines^21^. Depending on each patient’s individual needs, individual, self-managed and/or group training could be provided both in inpatient as well as in outpatient settings. In inpatient setting, team-based interventions, are provided 5 days a week and when needed also on weekends. Outpatient rehabilitation includes commonly interventions provided by physiotherapist and/or occupational therapists 1–3 times a week at primary care settings. The SALGOT experimental protocol was approved by the Swedish Ethical Review Authority (225-08) and written informed consent was obtained from all participants and/or their legal guardian(s) prior inclusion. All methods were performed in accordance with the Declaration of Helsinki. The Transparent Reporting of a multivariable prediction model for Individual Prognosis Or Diagnosis (TRIPOD) statement and the checklist for prediction model development was followed^22^.

### Clinical assessments

The Action Research Arm Test (ARAT) assesses the upper limb activity capacity and includes 19 items divided into four subscales (grasp, grip, pinch and gross movement) covering the main aspects of arm and hand use in daily activities^23,24^. Majority of items assess the ability to grasp and move objects of different shapes and sizes into different vertical or horizontal locations in the arms workspace. Each item is scored on a 4-point ordinal scale (0—can’t perform any part, 1—performs partly, 2—completes the task, but with abnormal movement components or body posture or the task takes more than 5 s but less than 1 min to complete, and 3—completes the task normally within the 5-s time limit). A maximum score of 57 indicates best performance. ARAT has excellent reliability, validity and responsiveness^25,26^. The reliability at item level have also been reported to be sufficient^26^.

The Fugl-Meyer Assessment of Upper Extremity (FMA-UE) assesses the upper limb motor impairment and includes 33 items divided into four subscales: shoulder/elbow, wrist, hand and coordination/speed^27^. Each item is scored on an ordinal 3-point scale, where 2 points are assigned when the movement is performed fully, 1 point when performed partially and 0 point when the movement cannot be performed. A total score of 66 indicates better motor function. The FMA-UE has excellent validity and is reliable both at the summed score and at item level^28–30^.

Grip strength was measured with the hydraulic hand dynamometer JAMAR (Sammons Preston, Chicago)^31^. The participants were seated with their arm in 90° of elbow flexion (antigravitational support was provided for the elbow position when needed by the tester) and instructed to squeeze the dynamometer with a maximum effort^32^. A mean of three trials was recorded (Pound force; 0–200) and the minimum readable value for grip force is 5 according to the manufacture. A mean value greater than 0 indicated a measurable grip strength. Grip strength measurement with the JAMAR dynamometer has proven to have excellent validity and reliability^31,33^.

The severity of stroke and initial arm paresis was determined by the National Institute of Health Stroke Scale obtained at admission^34^. Other clinical characteristics, the presence of sensation impairment, determined by the FMA-UE sensation, and the presence of spasticity of elbow and wrist joints determined by the Modified Ashworth Scale^35^ were collected for background data. Three experienced and trained physiotherapists, not involved in patient care, performed all clinical assessments. During an assessment session the protocols of the other assessment times were not available to the assessors.

### Selection of data for modelling

The original data set comprised of 122 participants, of these 6 died prior 3-months assessment and in 22 cases the 3-months outcome was missing due to different reasons described in Fig. 1. These data were removed and thereby 94 participants were retained in data modelling (Fig. 1). Baseline data was missing for 5 patients (4 missing ARAT, 1 missing grip strength, 1 missing FMA-UE). For these data points a single imputation was used to handle missing data within the data set. Mean was used for the grip strength and median was used for the remaining ordinal scales.

### The end-point outcome

The end-point outcome was defined as the arm activity capacity level, assessed by the ARAT, at 3-months post stroke. First, the full data set (n = 94) was plotted to explore potential clusters based on the ARAT total scores at baseline and end-point. An a priori K-means cluster analysis on the full dataset demonstrated five clusters in terms of 3-months functional outcome. The decision to limit to five clusters was made after using two statistical methods to define the optimal number of clusters (using Silhouette width and total width sum of squares). The final identified clusters according to 3-months ARAT scores were defined as poor (0–10 points), limited (11–32 points), good (33–50 points), excellent (51–56 points), and full outcome (57 points). These thresholds were similar to those published in several previous stroke cohorts^7–9^. Subsequently, two end-point cut-offs (ARAT ≤ 10 and ARAT ≤ 32) were tested for model development and performance. All five identified clusters were used to explore whether the final model could classify patients into the predefined clusters.

### Selection of independent variables

The independent variables were selected based on information published in the literature, suggesting that measures of grip, wrist or finger extension, and shoulder movement can provide good prediction for functional outcome^3,5–7,36^. The constraints were that the variables needed to be collected early after stroke (within 3-days post stroke), easily dichotomised, derived from an existing validated upper limb assessment test available in the SALGOT database (FMA-UE or ARAT, NIHSS arm) and were clinically implementable at bedside with relative ease. Items assessing distal hand and grip capacity along with items assessing proximal shoulder functions were considered essential parts for modelling. A model with fewer clinical variables were preferred over multiple variables to increase clinical applicability, but without sacrificing of predictive performance. Among single items of ARAT, the grasping of 2.5 cm cube was selected as first choice, partly because it represents an item with a middle range difficulty level according to Rasch analysis^15,37^ and partly because it can be performed at bedside. Single items of the FMA-UE shoulder and elbow subscale (Part A, volitional movements within and mixed synergies) and grip strength were also considered^5,6^.

For the analysis, the scores 0 and 1 of the ARAT 2.5 cm cube item were dichotomised as 0 (unable to complete the test within 1 min) and the scores 2 and 3 were assigned score 1 indicating ability to pick up a 2.5 cm cube with the more-affected hand alone and place it on a shelf (about the eyes height level) within 1-min time, irrespective to the grasp formation or compensation used. The FMA items score 0 was dichotomized as 0 (no active movement), and scores 1 and 2 were dichotomized as 1 (partly or full active movement). Grip strength, measured with Jamar hand dynamometer was dichotomised as 0 when unable to generate a measurable force and as 1 when able to generate a measurable force.

### Data analysis

Differences in clinical and demographic characteristics between the included (n = 94) and excluded (n = 28) patients were tested with either independent T-test, Mann Whitney U-test or Chi Square test.

**Figure 2:**
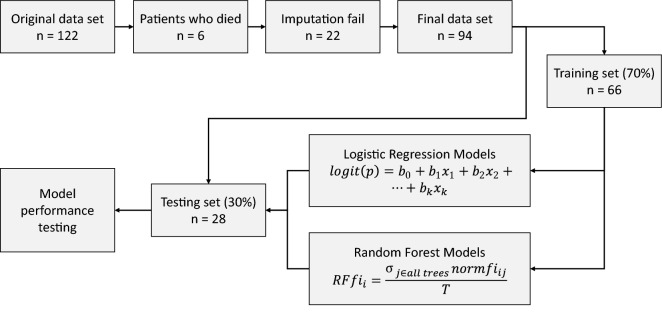
Flowchart over the modelling process.

As a first step, bootstrapping was used to generate 500 samples from existing dataset of 94 participants. During selection of independent variables, data was divided into two random statistically equivalent subsets, in which 70% (n = 66) was used in the training stage (Fig. 2). Forward stepwise logistic regression (LR) and random forest (RF) using the boosting method were used to select the independent variables for the prediction models^38^. RF classifiers^39^, are expected to have higher performance, higher accuracy and are more robust compared to regression and decision tree analysis^40,41^. The random forest (RF) machine learning embedded algorithms were used to determine the variable importance (entropy)^42^. Variables with explanatory power more than 10% were considered in further modelling. Both methods determine probabilities between zero and one. For the purposes of this study, the probability more than 0.5 was treated as favorable outcome and lower than 0.5 as not favorable outcome^40^.

The testing data set (n = 28) was used to evaluate the model performance. Sensitivity, specificity, kappa correlation coefficient and predicting accuracy were calculated for two functional outcome cut-offs (ARAT ≤ 10 and ARAT ≤ 32). Models including the total score of the FMA at 3-days post stroke and the arm sub-score of the NIHSS at admission were also evaluated for comparison to the models with short assessments alone. Kappa coefficients of 0.5 represents moderate agreement, above 0.7 good agreement and 0.8 excellent agreement^43^.

Multinomial logistic regression was used as extension to binary logistic regression to predict probability of the category membership on 5-level outcome. The logistic coefficient (B), odds ratio and 95% coefficient intervals were calculated for each independent variable for each alternative category of the outcome variable. Maximum likelihood ratio estimation and Chi-square test were used to evaluate the probability of categorical membership.

Using the information gleaned from the regression models, decision support algorithms were developed to explore if simple algorithms could be developed to predict the functional outcome profile of stroke patients. The misclassified cases in the poor and limited outcome groups were further explored by using the available assessments at 10 days and 4 weeks post stroke along with other clinical characteristics.

## Results

The demographic and clinical characteristics of the original non-selected SALGOT cohort (n = 122), the final dataset (n = 94) included in the analyses and the excluded group with missing data at 3-months are shown in Table 1. The excluded group (n = 28) had a more sever stroke assessed by NIHSS, higher proportion of individuals with total anterior circulation infarct and lower upper limb function assessed by FMA-UE 3-days post stroke.

**Table 1:** Baseline demographic and clinical data for all patients, patients who survived and patients who informed the modelling process.

Characteristics Mean (SD) or n (%)	SALGOT cohort n = 122	Included in the prediction modelling n = 94	Excluded from modelling n = 28
Age, years, mean (SD)	69.6 (12.9)	68.4 (12.5)	73.4 (13.8)
**Sex**			
Female	54 (56%)	54 (57%)	14 (50%)
Male	68 (44%)	40 (43%)	14 (50%)
**Stroke type**			
Haemorrhage	19 (16%)	17 (18%)	2 (7%)
Infarct	103 (84%)	77 (82%)	26 (93%)
**Type of ischemic stroke**			
Total anterior circulation infarct	15 (14%)	6 (8%)^a^	9 (35%)^a^
Partial anterior circulation infarct	44 (43%)	34 (44%)	10 (38%)
Lacunar infarct	36 (35%)	30 (39%)	6 (23%)
Posterior circulation infarct	8 (8%)	7 (9%)	1 (4%)
**Paretic arm**			
Right	56 (46%)	40 (43%)	16 (57%)
Left	66 (54%)	54 (57%)	12 (43%)
**Stroke severity at admission, median (Q1, Q3)**	7 (3, 14)	6 (3, 11)^a^	11 (4, 18)^a^
Mild (NIHSS 0–4)	42 (36%)	36 (40%)	6 (23%)
Moderate (NIHSS 5–15)	49 (43%)	40 (45%)	9 (35%)
Severe (NIHSS ≥ 16)	24 (21%)	13 (15%)^a^	11 (42%)^a^
**NIHSS Arm at admission, median (Q1, Q3)**	2 (1, 4)	2 (1, 4)	3 (1, 4)
0 (no drift)	16 (14%)	14 (16%)	2 (7%)
1–3	63 (54%)	50 (56%)	13 (49%)
4 (no movement)	37 (32%)	25 (28%)	12 (44%)
Thrombolysis	15 (12%)	9 (10%)	6 (21%)
Thrombectomy	5 (4%)	3 (3%)	2 (7%)
Days at stroke unit, mean (SD)	13.7 (8.5)	12 (6.9)	19.5 (10.7)
**Ongoing rehabilitation at 3 months**			
Inpatient	6 (6%)	6 (6%)	No data
Outpatient	49 (52%)	48 (51%)	No data
**Clinical characteristics at 3 days post stroke**			
FMA-UE, mean; median (Q1, Q3)	29.8 (25.2) 21 (4, 57)	33.1 (24.9) 39 (4, 58)^a^	18.9 (23.3) 4 (1, 41)^a^
Sensory impairment (FMA-UE < 12)	65 (53%)	45 (48%)	20 (71%)
Spasticity (mAS ≥ 1),	28 (23%)	22 (23%)	6 (21%)
**Clinical characteristics at 3 months**			
FMA-UE, mean (SD), median (Q1, Q3)	48.4 (22.4) 61.5 (33, 66)	48.4 (22.4) 61.5 (33, 66)	No data
Sensory impairment (FMA-UE < 12)	25 (27%)	25 (27%)	No data
Spasticity (mAS ≥ 1)	32 (34%)	32 (34%)	No data

^a^Statistically significant difference between included and excluded patients (Chi-Square or Mann Whitney U-test, p < 0.05). *NIHSS* National Institute of Health Stroke Scale, *FMA* Fugl-Meyer Assessment, *mAS*, modified Ashworth Scale.

### Prediction algorithm

Several preliminary models using both logistic regression and random forest were calculated. The best performing model according to accuracy and agreement estimates, comprising 3 short clinical assessments collected within 3-days post stroke, included ARAT cube 2.5 cm, grip strength and FMA shoulder elevation and/or abduction within synergies. The classification accuracy and agreement estimates for the two cut-offs (ARAT ≤ 10 and ARAT ≤ 32) at 3-months are shown in Table 2. Overall, the logistic regression showed a better performance compared to random forest analysis. High sensitivity (0.96), specificity (0.92) prediction accuracy (0.94) and excellent Kappa agreement (0.87) were reached for the ARAT ≤ 10 cut-off. The sensitivity (0.88), specificity (0.82), accuracy (0.86) were somewhat lower for the ARAT ≤ 32 cut-off and the Kappa agreement was moderate (Kappa 0.68). The additional models that also included FMA total score from day 3 post stroke and/or NIHSS arm score at admission showed lower (NIHSS Arm alone) or comparable (FMA total alone or combined with NIHSS Arm) model performance for differentiation between poor and limited cut-off compared to the models that only included short assessments (Supplementary Table 1).

**Table 2:** Model performance determined by logistic regression (LR) analysis and Random Forest (RF) modeling for the two ARAT cut-offs between the poor, limited and good functional outcome.

Thresholds for ARAT at 3 months	Final models	Sensitivity	Specificity	Kappa	Accuracy
0 = ARAT ≤ 10 1 = ARAT ≥ 11	D3 cube 2.5 D3 grip strength D3 FMA A.II elevation D3 FMA A.II abduction	0.96 (LR) 0.81 (RF)	0.92 (LR) 0.83 (RF)	0.87 (LR) 0.58 (RF)	0.94 (LR) 0.82 (RF)
0 = ARAT ≤ 32 1 = ARAT ≥ 33	D3 cube 2.5 D3 grip strength D3 FMA A.II elevation D3 FMA A.II abduction	0.88 (LR) 0.82(RF)	0.82 (LR) 0.84 (RF)	0.68 (LR) 0.64 (RF)	0.86 (LR) 0.81 (RF)

*ARAT* Action Research Arm Test, *FMA* Fugl-Meyer Assessment, *LR* logistic regression, *RF* random forest.

**Figure 3:**
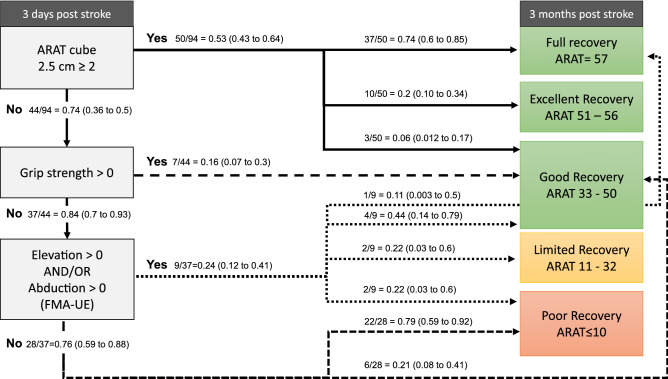
Conditional probability tree algorithm along with 95% confidence intervals for the 5 functional outcome strata according to Action Research Arm Test assessed at 3-months after stroke.

The observed and predicted frequencies for multinomial outcome at 3-months are shown in Table 3. The overall model fit was statistically significant (Chi-square 31.57, p < 0.001). The logistic coefficient (B), odds ratio with 95% coefficient intervals for each independent predictor variable are shown in Supplementary Table 2. The results showed that the ability to grasp and lift an ARAT cube of 2.5 cm was a good predictor for excellent and full functional outcome category, whereas a poor motor performance within 3-days post stroke (ARAT cube, grip strength and motor function in shoulder elevation/abduction all 0) identified those who were unlikely to have good recovery (Table 3). Differentiation was uncertain for the middle categories due to the small number of observations in the study population. However, ability to produce some grip force within 3-days post stroke was indicative for at least good functional outcome while the absence of voluntary motor activity in in shoulder elevation or abduction within 3-days post stroke indicated that excellent or full functional outcome is rather unlikely. The grip strength and ARAT cube showed to be the most important independent variables for the prediction of limited and good level of functional outcome, respectively (Supplementary Table 2). The developed prediction algorithm based on the results from regression analysis along with conditional probabilities is shown in Fig. 3.

**Table 3:** Observed and predicted frequencies for prediction algorithm.

Observed and predicted frequencies								
Motor function within synergies (FMA-UE) Day 3	Grip strength Day 3	ARAT cube 2.5 cm Day 3	Multinomial outcome ARAT at 3 months	Frequency			Percentage	
				Obs	Pred	Pearson residual	Obs	Pred
Elevation = 0 AND/OR Abduction = 0	Grip < 0	Cube < 2	Poor	22	23.217	− 0.611	78.6%	82.9%
			Limited	0	0.084	− 0.290	0.0%	0.3%
			Good	6	2.681	2.131	21.4%	9.6%
			Excellent	0	0.420	− 0.653	0.0%	1.5%
			Full	0	1.597	− 1.301	0.0%	5.7%
Elevation > 0 AND/OR Abduction > 0	Grip < 0	Cube < 2	Poor	2	8.800	− 15.384	22.2%	97.8%
			Limited	2	0.001	82.557	22.2%	0.0%
			Good	4	0.193	8.756	44.4%	2.1%
			Excellent	0	0.000	0.000	0.0%	0.0%
			Full	1	0.006	12.753	11.1%	0.1%
	Grip > 0	Cube < 2	Poor	0	0.069	− 0.265	0.0%	1.0%
			Limited	0	0.006	− 0.076	0.0%	0.1%
			Good	7	6.786	0.470	100.0%	96.9%
			Excellent	0	0.029	− 0.170	0.0%	0.4%
			Full	0	0.110	− 0.334	0.0%	1.6%
		Cube ≥ 2	Poor	0	3.174	− 1.841	0.0%	6.3%
			Limited	0	0.265	− 0.516	0.0%	0.5%
			Good	3	3.688	− 0.372	6.0%	7.4%
			Excellent	10	9.275	0.264	20.0%	18.6%
			Full	37	33.598	1.025	74.0%	67.2%

The percentages are based on total observed frequencies in each subpopulation.

*ARAT* Action Research Arm Test, *FMA* Fugl-Meyer Assessment.

### Additional explorative analysis

In the current dataset, 6 participants with predicted poor functional outcome and 5 participants with limited outcome actually showed good recovery (Table 4). Furthermore, 2 participants with predicted limited outcome showed instead poor recovery. The grip strength assessment at 10 days and 4 weeks as well as stroke type were identified as potential variables that could explain the misclassification and refine the overall prediction. All 11 participants who showed better recovery than predicted and had initial poor or limited outcome prediction showed measurable grip strength at 4 weeks. In contrast, the 2 participants who showed a worse recovery than predicted at 3-days post stroke showed no measurable grip strength at 10 days nor 4 weeks post stroke. In addition, in the group who showed better than expected recovery, 7 out of 11 (64%) had haemorrhagic stroke in contrast to 10 out of 81 (12%) among those with correct prediction. The 2 participants with worse than expected recovery outcome both had ischemic stroke.

**Table 4:** Grip strength measured at 10-days and 4-weeks post stroke shown in patients with poor or limited predicted functional outcome.

		Actual outcome			Grip strength > 0		
		Poor	Limited	Good	Day 3	Day 10	Week 4
Predicted outcome	Poor	22			0	0/22	3/22
				6	0	2/6	6/6
	Limited	2			0	0/2	0/2
			2		0	0/2	2/2
				5	0	2/5	5/5

Values indicate frequencies (n).

## Discussion

The results of this study showed that prediction of upper limb activity capacity at 3-months post stroke can accurately be done as early as 3-days post stroke by using clinically feasible assessments. The final prediction model included 3 simple bedside assessments applied in a hierarchical order: (i) ability to grasp, lift up and release a small wooden cube approximately at eye height level (ARAT Grasp item); (ii) ability to produce any measurable grip strength (Jamar hand dynamometer); and (iii) ability to show at least some active proximal upper limb movement (shoulder abduction and/or shoulder elevation within flexor synergy) according to FMA.

In clinical practice, early prediction of motor outcome is necessary to select most appropriate intervention for a specific patient as well as to plan discharge and continuing rehabilitation. A patient with expected good recovery will need intensive early rehabilitation with focus on movement quality to regain movement patterns as close to pre-stroke patterns as possible. On the other hand, a patient with expected limited recovery, where the improvements are expected to be slower, strategies are need to prevent further deterioration (e.g. atrophy and learned non-use) and the use of technology-based rehabilitation may be required. It may also be that compensatory movement strategies need to be learned earlier when expected recovery is limited or delayed in order to regain independence in activities of daily living (ADL). Taken these two main rehabilitation approaches, regaining movement patters similar to pre-stroke condition or introducing technology solutions and compensatory movement strategies, it becomes clear that differentiation between good and poor or limited recovery will have the uppermost importance for clinical decision making. The findings of the current study showed that the proposed prediction algorithm had high sensitivity, specificity and accuracy to differentiate poor functional outcome (0.92–0.96) and poor and limited outcome (0.82–0.86) from the good outcome categories and can therefore be used for these purposes.

Motor impairment and severity of initial upper limb paresis is one of the strongest predictors of functional outcome in people with stroke^3,6,44^. Prediction is more accurate in patients with moderate or mild initial motor impairment compared to severe initial impairment^11,17,36^. It is also worth to notice that even when the clinical scales alone perform well at the group level, recovery pathways at individual may still vary^8^. An accurate prediction becomes particularly important for patients who despite predicted poor outcome will over time regain motor function that will allow them to use the paretic arm in their routine daily activities. In these patients, the compensatory movement strategies should be kept minimal early on to avoid learned non-use and complemented with retraining of active-assisted movement control of the paretic arm. In our data, every fifth patient (6 out of 28) with initial poor motor function and expected poor outcome, actually reached a good functional outcome at 3-months post stroke. All these 6 patients had a measurable grip strength present at 4 weeks post stroke. This finding suggests that a simple measurement of grip strength might be used to revaluate and refine the prognosis and to adjust treatment content for patients with initial poor motor function. It also suggests that rehabilitation interventions preventing secondary complications such as decreased range of motion and pain need to be included in the rehabilitation regime in patients with initial limited motor function to facilitate potential delayed recovery. For example, poor motor function, reduced range of motion and pain have shown to be associated with post stroke spasticity^45^, but the likelihood to develop contractures was highest for those who did not gain motor function within the first 6 weeks of stroke^46^.

Our data also revealed that 64% of patients showing better than expected recovery had a haemorrhagic stroke compared to 12% in the group with correct prediction. This finding is in line with previous research showing different recovery patterns in patients with ischemic and haemorrhagic stroke^47^. In haemorrhagic stroke, the initial upper limb motor impairment was worse compared to the ischaemic stroke, but at 3-months post stroke no difference in motor function was detected between the two groups^47^. These findings suggest that in order to provide accurate outcome prediction for patients with limited initial motor function regular follow-up assessments during the first months are particularly important for patients with haemorrhagic stroke.

In addition to clinical assessments, neurophysiological and neuroimaging techniques, used to determine the corticospinal integrity, have shown to be useful particularly to improve prediction accuracy in patients with poor initial motor function^1,10,14^. Clinical implementation of these advanced techniques has, however, been restricted due to the limited availability and expertise to run and integrate the results into clinical decision-making processes^9^. The second Predicting Recovery Potential (PREP2) algorithm that combines simple clinical assessments and determination of motor evoked potentials (MEPs) using transcranial magnetic stimulation is so far the only model that has been implemented in clinical settings and that also has shown impact on clinical decision making^13^. After implementation the length of hospital stay was shortened by 1 week, the therapists reported a higher confidence regarding expected outcome and the content of therapy was modified according to prediction. Despite this excellent example, the prediction algorithms including simple clinical data will have an advantage over the more complex models when implemented in clinical practice. Therefore, there is an urgent need to implement and evaluate the usefulness and efficacy of these clinical prediction models in clinical settings as well. Recently, a prediction algorithm only including clinical bedside assessment from the PREP2 algorithm reported an overall accuracy of 61% at predicting upper limb activity capacity at 3 months post stroke, although the sensitivity and specificity varied across the four outcome categories^17^. The authors concluded that the model was overall better than change for each four outcome categories and could be implemented in clinical practice with a reservation that individuals with poor initial motor function might need repeated assessments to refine prediction^17^.

Recently a complex computerized longitudinal prediction model was proposed that comprises input of repeated clinical assessments of FMA-UE^11^ and ARAT^48^. The overall accuracy for predicting poor, moderate and good recovery at 3- and 6-months was around 0.80^11^. A larger prediction error was noted for patients with low initial score compared to patients with higher scores, although the prediction errors decreased with an increased number of repeated assessments included in the modelling^11,48^. This work is promising and in line with consensus-based recommendations for clinical assessments in stroke rehabilitation^49–51^. The short length of stay in stroke units^52,53^ and many other assessments that need to be done early after stroke might, however, hinder implementation and compliance of this model. A need to enter test results on a customized platform or webpage will add administration time and further hamper successful implementation in clinical practice. Even when the use of longer comprehensive assessments early after stroke need to be justified, so that the extra time and effort will produce added value, these more comprehensive assessments like FMA and ARAT are needed to aid intervention selection and evaluation of outcome during the whole recovery process.

Several demographic and clinical factors like age, sex, stroke type, affected side, hand dominance, intravenous thrombolysis have not shown added value in prediction models when motor function is already included^3,6,9,11,48^. Age was, however, included as factor in the PREP2 algorithm to refine differentiation between excellent and good outcome groups^9^. Modelling of PREP2 algorithm also showed that FMA-UE and SAFE score had similar prediction accuracy, and therefore the shorter assessment of SAFE was selected. These examples provide evidence that a simple model, with few key clinical assessments of motor function could be as good alternative than a more complex model.

Prediction models and interpretation of the results need to be meaningful for the patient and clinician. The dichotomized binary outcomes of good or poor outcome have been criticized, since they will only provide limited information^54^. Having ability to extend fingers and abduct shoulder 2 days after stroke indicated a 98% probability of achieving at least 10 points on the ARAT at 6 months^7^. This simple prediction model increased the awareness of how simple clinical tests can be utilized, but the practical usefulness of this model remains limited due to the wide range of functional recovery possible between the 10 and 57 points of ARAT^9^. Here the development of PREP algorithm has proved that differentiation of end-point outcome with different levels will improve the clinical usefulness of prediction^8,9^. For example, clinically meaningful endpoints evaluated in a large stroke population showed that shoulder abduction, finger and elbow extension predicted ability to use the arm at least in basic ADL (FMA-UE more than 32 points), while wrist extension and supination predicted the use the arm in routine ADL 6 months post stroke (FMA-UE more than 57 points)^6^.

Prediction algorithms applied early, and preferably within the first week after stroke onset are most useful for rehabilitation and discharge planning. Early prediction is also important when considering the constantly decreasing length of hospital stay and improved acute care^55^. The median time in stroke unit was reported to be 7 days in Sweden and 2–8 days in Australia in 2019^52,53^. These numbers point out the need to implement simple and informative prognosis indicators during the first days after stroke onset. These indicators can be used to crudely identify patients with favourable functional recovery who will most likely have a short hospital stay. Patients with less favourable initial prognosis will most likely need a longer rehabilitation and then the repeated assessments might provide a refined estimate for long-term recovery. For the endpoint, a specified time of 3-, 6- or 12-months after stroke onset is recommended over the discharge time for prediction models^50,54^. Most of the recovery and also the rehabilitation interventions are concentrated to the first 3-months post stroke, which makes this time-point relevant for prediction. However, it is important to notice that continuous functional improvements can also be regained after this time. Furthermore, when a prediction model will be implemented in clinical practice, appropriate training and support need to be provided to clinicians to safely deliver predictions to patients and families.

### Strengths and limitations

An unselected SALGOT stroke cohort recruited early at stroke unit composed the original dataset of this study. The demographic and clinical characteristics, such as age, stroke type, stroke severity and endovascular treatment of the original SALGOT dataset are well in line with typical patient cohorts in acute stroke. In the prediction modelling, patients with missing data at 3-months endpoint were excluded for different reasons (declined, death, not able to follow instructions, recurrent stroke). The subsequent analysis showed that the excluded subgroup had more severe stroke and lower motor function 3-days after stroke compared to the final dataset. This means that the findings of the current study are most applicable for patients with potential to survive and be assessed at 3-months post stroke. Future studies are warranted to externally validate the proposed prediction algorithm in a separate independent dataset.

A limitation of the study was that the potential predictor variables were selected among available assessments from the SALGOT-study. However, knowledge from previously developed prediction models guided the variable selection^3,5–7,36^. Taken that, potential predictor variables assessing distal (hand and grip) and proximal (shoulder) upper limb function as well as grip strength and stroke severity (NIHSS) were considered. It should be noted that all assessments included in the prediction model were collected in a standardized way. If the patients could not leave the hospital room, the assessment at 3 days post stroke was performed bedside (sitting upright on the side of the bed or on a chair beside the bed). The use of standardized equipment such as hand dynamometer or standardized height shelf (ARAT cube) might be a limitation for clinical implementation. On the other hand, the ARAT cube item could easily be adapted to clinical settings and instead of using a shelf, the height of the lift and release of the cube could be assessed as a lift and release at the tested person’s eye level. Validity of this adaptions need however to be investigated in a separate cohort.

A comprehensive data modelling process was used in this study, which strengthens the results. The prediction modelling was first done in the training subsets of data (70%) and subsequently validated in the testing subtest (30%). An algorithm randomly assigned cases to each dataset and therefore identification of cases in each dataset was not possible. A large number of potential predictors were considered and tested to reach the final set of predictors and two statistical analysis methods (logistic regression and random forest) were used. Due to the low number of observations available for prediction of the middle categories of functional outcome, these results need to be confirmed in a larger dataset.

### Clinical implications and conclusions

The results of the current study demonstrate that simple clinical assessments performed at 3-days post stroke can successfully be used to predict the upper limb activity capacity level at 3-months after stroke onset. In patients with poor initial motor function (no ability to grasp or move the arm and shoulder against gravity) and particularly in those with haemorrhagic stroke a follow-up assessment of grip strength during the first 4 weeks post stroke is recommended to refine the initial prognosis.

The suggested prediction model, only including simple bedside clinical assessments, can potentially be used in any acute stroke unit around the world. For example, the prediction algorithm might facilitate and inform selection of treatment approach. For patients with good expected recovery active functional treatments can be introduced early with higher confidence, while for patients with expected limited or delayed recovery interventions reinforcing independence in ADL and preventing secondary complications could be in focus. Although external validation of this proposed tool is needed before clinical use.

## Supplementary Information

Supplementary Information 1.

Supplementary Information 2.

## Data Availability

Anonymized data included in the prediction modelling will be made available by request from any qualified investigator to the first corresponding author (margit.alt-murphy@neuro.gu.se) with a requirement of an approved permission from the Swedish Ethical Review Board.
