# Supplementary material for: Early prediction of upper limb functioning after stroke using clinical bedside assessments: a prospective longitudinal study
Source: Sci Rep. 2022 Dec 21;12:22053. doi: 10.1038/s41598-022-26585-1 (PMC9772392; doi:10.1038/s41598-022-26585-1)
Supplement: Supplementary file 1 — Supplementary Information 1. [file 41598_2022_26585_MOESM1_ESM.docx]

**Supplementary table 1.** Performance of the final and additional logistic regression models for predicting 3 months outcome between poor, limited and good functional outcome at 3 months.

| **Thresholds for ARAT at 3 months**  **Additional models** | **Sensitivity** | **Specificity** | **Kappa** | **Accuracy** |
| --- | --- | --- | --- | --- |
| **0 = ARAT ≤ 10; 1 = ARAT ≥ 11** | | | | |
| **Day 3 cube 2.5; Day 3 grip strength**  **Day 3 FMA A.II elevation; Day 3 FMA A.II abduction** | **0.96** | **0.92** | **0.87** | **0.94** |
| Day 3 cube 2.5; Day 3 grip strength  D3 FMA Total | 0.95 | 0.80 | 0.75 | 0.92 |
| Day 3 cube 2.5; Day 3 grip strength  NIHSS Arm admission | 0.75 | 60 | 0.28 | 0.72 |
| Day 3 cube 2.5; Day 3 grip strength  Day 3 FMA Total; NIHSS Arm admission | 1 | 0.90 | 0.92 | 0.96 |
| **0 = ARAT ≤ 32; 1 = ARAT ≥ 33** | | | | |
| **Day 3 cube 2.5; Day 3 grip strength**  **Day 3 FMA A.II elevation; Day 3 FMA A.II abduction** | **0.88** | **0.82** | **0.68** | **0.86** |
| Day 3 cube 2.5; Day 3 grip strength  D3 FMA Total | 0.91 | 0.91 | 0.85 | 0.92 |
| Day 3 cube 2.5; Day 3 grip strength  D3 NIHSS Arm at admission | 0.44 | 0.75 | 0.42 | 0.67 |
| Day 3 cube 2.5; Day 3 grip strength  Day 3 FMA Total; NIHSS Arm admission | 0.91 | 0.91 | 0.85 | 0.92 |

The final models used in prediction algorithm are marked bold. Abbreviations: ARAT, Action Research Arm Test; FMA, Fugl-Meyer Assessment; NIHSS, National Institute of Health Stroke Scale

**Supplementary Table 2.** Results from the multinomial logistic regression for each independent variable.

| **Parameter Estimates** | | | | | | | | |
| --- | --- | --- | --- | --- | --- | --- | --- | --- |
| Actual recovery | | B | Std. Error | Wald | Sig. | Exp(B)  OR | 95% CI Exp(B) | |
|  |  |  |  |  |  |  | Lower Bound | Upper Bound |
| Poor | Intercept | -2.451 | .610 | 16.122 | .000 |  |  |  |
|  | [Grip strength=.00] | 1.311 | 5.021 | .068 | .794 | **3.71** | .000 | 69708.79 |
|  | [Cube 2.5=.00] | 1.949 | 4.983 | .153 | .696 | **7.02** | .000 | 122535.16 |
|  | FMA-elev/abd =.00] | 1.951 | .000 | . | . | **7.04** | 7.037 | 7.04 |
| Limited | Intercept | -4.894 | 1.994 | 6.024 | .014 |  |  |  |
|  | [Grip strength=.00] | 46.900 | 14.093 | 11.075 | **.001** | **2.33E-20** | 2.36E-8 | 2. 31E-32 |
|  | [Cube 2.5=.00] | 1.949 | 13.727 | .020 | .887 | **7.02** | 1.45E-11 | 3.40E-12 |
|  | FMA-elev/abd =.00] | -46.900 | .000 | . | . | **4.283E-21** | 4.28E-21 | 4.28E-21 |
| Good | Intercept | -2.213 | .548 | 16.327 | .000 |  |  |  |
|  | [Grip strength=.00] | -5.860 | 3.230 | 3.292 | .070 | **.003** | 5.08E-6 | 1.60 |
|  | [Cube 2.5=.00] | 6.350 | 3.110 | 4.170 | .041 | **572.46** | 1.29 | 253900.44 |
|  | FMA-elev/abd =.00] | 2.416 | .000 | . | . | **11.21** | 11.206 | 11.21 |
| Excellent | Intercept | -1.289 | .370 | 12.137 | .000 |  |  |  |
|  | [Grip strength=.00] | -14.786 | 6.904 | 4.587 | **.032** | **3.789E-7** | 5.03E-13 | .285 |
|  | [Cube 2.5=.00] | -.046 | 6.664 | .000 | .994 | **.955** | 2.03E-6 | 4.49E-5 |
|  | FMA-elev/abd =.00] | 14.786 | .000 | . | . | **2.639E-6** | 2.64E-6 | 2.64E-6 |
| The reference category is: full recovery. | | | | | | | | |

Abbreviations: FMA, Fugl-Meyer Assessment
